# The intriguing possibility of using probiotics in allergen-specific immunotherapy

**DOI:** 10.1016/j.waojou.2023.100751

**Published:** 2023-02-19

**Authors:** Fabiana Furci, Marco Caminati, Ernesto Crisafulli, Gianenrico Senna, Sebastiano Gangemi

**Affiliations:** a Asthma Centre and Allergy Unit, University of Verona and Verona University Hospital, Verona, Italy; b Allergy, Asthma and Clinical Immunology, Department of Medicine, University of Verona and Verona University Hospital, Verona, Italy; c Respiratory Medicine Unit and Section of Internal Medicine, Department of Medicine, Verona University Hospital, Verona, Italy; d School and Operative Unit of Allergy and Clinical Immunology, Department of Clinical and Experimental Medicine, Policlinico “G. Martino”, University of Messina, Messina, Italy

**Keywords:** Allergen immunotherapy, Microbiome, Microbiota, Probiotics, Respiratory allergies

## Abstract

Allergen immunotherapy (AIT) can be considered the etiological therapy for allergic rhinitis and hymenoptera venom allergy. Its role is increasingly emerging in the context of IgE mediated food allergy, where the achievement of tolerance, or the permanent resolution of an allergy, represents the optimal goal of AIT.

AIT treatment, indicated in adults and children with allergic rhinitis, has a preventative effect on the development of asthma and can also be used when asthma is associated to rhinitis; however, it is not the first choice for treatment of isolated asthma.

While knowledge on immunological mechanisms, efficacy, and safety of AIT is known, an intriguing line of investigation has arisen on how the action of AIT is modulated by the use of probiotics, starting from awareness that the microbiome is altered in allergic conditions: the use of probiotics in inducing the stimulation of innate immunity via toll-like receptor activation, thus acting as adjuvants in AIT, is hereby examined.

Therefore, by analyzing literature on AIT and probiotics, we intend to draw attention to how the role and use of AIT are emerging as being increasingly important for both the short- and long-term management of allergic diseases and how recourse probiotics may represent an additional therapeutic strategy to modulate the effectiveness of AIT. However, further investigations are needed to better identify which probiotics to use, the dosage, and the optimal duration to obtain correct immunomodulation, and how to best customize their use, including a “AIT + probiotics” strategy in the field of precision medicine.

## Introduction

Allergen immunotherapy is the only specific, disease-modifying therapy for most IgE allergic diseases, in particular allergic rhinitis (AR) and/or allergic asthma, and patients with anaphylactic reactions to Hymenoptera venom.^1^ It consists of the administration of the culprit allergen by subcutaneous injections or by sublingual administration and can be considered a cornerstone in the treatment of allergic disorders.

Clinical efficiency of AIT is based on immunologic mechanisms that promote regulatory T cells (Tregs) and downplay the immune response caused by allergens, inducing specific tolerance beyond duration of the treatment and preventing the development of new allergic disorders.^1^, ^2^, ^3^ The concept of immune tolerance induced by AIT consists of the induction of long-term unresponsiveness to allergens either in natural exposure settings or in *in vivo* challenges. This is the result of the action of immune cells, tissues, and mediators, with changes in allergen-specific memory T- and B-cell responses, diminished IgE, improved IgG4 production from B cells, and downregulation of mast cell and basophil activation, with suppression of allergic symptoms.^4^^,^^5^

Moreover, considering the mechanisms of AIT, it should be noted that Tregs are important regulators for immunological mechanisms in peripheral tolerance to allergens. The switch from allergen-specific effector T cells to a regulatory phenotype is a significant step for the efficacy of AIT. The induction of a tolerant state in peripheral T cells represents an essential effect of AIT and is characterized by the generation of allergen-specific Tregs that produce IL-10 and TGF-β, and by the suppression of allergen-specific Th2 and Th1 cells. This concept is highlighted by the feedback of local Foxp3+CD25^+^CD3^+^ cells in the nasal mucosa, their increase after immunotherapy, and association with clinical efficacy and improvement of allergic rhinitis.^6^

Duration of AIT is important: 3 years of subcutaneous immunotherapy (SCIT) or sublingual immunotherapy (SLIT) is reported to be clinically successful for AR, modulating allergic immune responses toward a 2-3-year sustained tolerant state, following termination of the therapy. AIT is known to provide a long-term clinical benefit that may persist for several years after cessation of treatment.^1^

Along with the progression of research to improve the efficacy of AIT, there is an increasing interest in the role of the microbiome in modulating the immune system and in its possible role in modulating the efficacy of AIT.^7^ The microbiota, which is composed of trillions of microbial organisms, including bacteria, fungi, and viruses, can be considered our largest organ with significant emerging roles in immune regulation, cancer development control, and bone remodeling. During the last decade, the alteration of the microbiota (dysbiosis) has been linked to various disorders.^8^^,^^9^

In this paper, we aimed to analyze data reported in literature regarding the possible immuno-modulating role of probiotics during AIT, in patients affected by IgE-mediated allergic disorders.

## Microbiota, probiotics and allergic disorders

In 2010, the first study regarding the lung microbiome in asthma and healthy controls was publishe; before this date the lower airways were considered sterile.^10^, ^11^, ^12^ The upper respiratory airways in childhood asthma are colonized by *Haemophilus influenza, Moraxella catharralis,* *and Streptococcus* *spp* that are associated with increased *ex vivo* Th2 cytokines such as IL-5, IL-13, and IL-17.^13^^,^^14^ *Mycoplasma pneumoniae, Chlamydophila pneumoniae, Chlamydia tracomatis, Staphylococcus aureus,* and *Haemophilus influenza* have been associated with to severe asthma and asthma exacerbations, independent of the use of inhaled corticosteroids. Indeed, an increased presence of *Actinobacteria* has been associated with better asthma control.^15^ Several phyla, highlighted in the airway flora of asthmatic and non-asthmatic patients, play an immune-modulating role on mucosal immunity, likely favoring asthma prevention. In particular, patients with asthma have a greater presence of *Proteobacteria*, associated with poor control of the disease; patients not affected by asthma are colonized predominantly by beneficial phyla, such as *Firmicutes* and *Actinobacteria.*^15^ In the same way, for food allergies, as reported in some studies, considering that children affected by food allergy have distinct gut microbiome compositions compared to children not affected by food allergy, supplementation with specific "missing" immunomodulatory microbes could represent a novel approach to the primary prevention of many human atopic diseases.^16^^,^^17^ Gastrointestinal microbiota modifications may interrupt mucosal immunological tolerance, inducing allergic conditions such as food allergy. The interruption of tolerance is achieved mainly through the action of gut microbiota, its metabolic products, epithelial cells, IgA antibodies, and Tregs.^18^

Bacterial therapies, such as probiotics, have been studied for the treatment of allergic disorders, but there are some limitations due to the heterogeneity of studies and variable outcome measures. Probiotics play a relevant role in eliciting Th1 cytokine production, inducing Tregs, suppressing Th2 pathways. *Lactobacillus* bacteria play a crucial role in Tregs inducing the generation of semi-mature dentritic cells (DC) and increasing the expression of CD40; moreover, they have a role in inhibiting IL-4 and IL-5, favoring IL-10 and TGF- β. Probiotics improve local IgA production, which has a significant role in mucosal defenses.^9^^,^^18^

Some studies have considered the immunomodulatory role of probiotic supplementation in promoting tolerance, acting with an anti-inflammatory role, reducing the risk of accumulation of inflammatory molecules, such as reactive oxygen species (ROS). In particular, probiotics have balancing properties between TH1 and Th2 responses, regulating the production of pro-inflammatory and anti-inflammatory cytokines, also modifying genes during inflammation, influencing the morphology and target of cells. DCs, antigen presenting cells (APCs), play a relevant role in the identification and selection of pathogens between commensal bacteria and probiotics. In this identification, toll-like receptors (TLR) have a key function.^19^

As reported by Thaiss CA et al, the possible immunological use of probiotics for the prevention of allergic disorders is based on the activation of TLR2 on intestinal epithelial cells with a reduction of gut permeability, the activation of TLR2 on myeloid DCs inducing differentiation into tolerogenic DCs and the activation of Treg cells. This latter activation is also induced by the activation of TLR9 on differentiated tolerogenic DCs. Therefore, the role of probiotics can modulate various crucial immunological factors, the activation of tolerogenic immune responses, the suppression of inflammatory intestinal response, and/or the promotion of the integrity of the gut barrier with the induction of IgA production.^20^

Emerging evidence reported that the increase in food allergy (FA) over the past few decades was associated with changes in the diversity and composition of the gut microbiota. In particular recent research highlighted that short-chain fatty acids (SCFAs), the main metabolites derived from gut microbiota, contributed to FA protection.^21^

Some studies reported that the SCFAs concentration of FA patients was significantly lower than that of control groups.^22^ Current studies reported that SCFAs, in particular acetate, propionate and butyrate, about 90% of SCFAs produced by gut microbiota, were associated with the protection against FA.^21^ Human clinical studies reported that the intake of acetate during pregnancy was linked to a reduced occurrence of FA in offspring.^23^ Roduit et al^24^ analyzing SCFAs levels in 301 fecal samples of 1-year-old children reported that propionate and butyrate levels were negatively correlated with atopic sensitization and future allergic diseases. Cait et al^25^ observed that butyrate produced in early infancy was related to immune tolerance and the infants with allergic sensitization in late childhood lacked genes encoding key enzymes for the production of butyrate. Therefore, increasing evidence from human and mouse models reported that signals derived from gut microbiota metabolites SCFAs in the lumen of the intestine acted as key regulators of FA. The function of multiple types of immune cells was affected directly or indirectly by SCFAs through different mechanisms and pathways in intestinal immunity. In particular, many potential mechanisms of SCFAs are involved in the regulaton of the immune system in FA: fortifying the intestinal barrier integrity by the receptors on the surface of the intestinal epithelial cells (IECs), inducing the FA alleviation by acting on IECs, ILCs, DCs, T and B cells, or reducing inflammatory response by acting on mast cells.^21^

About food allergy treatment the induction of Tregs has been a topic of great interest. B cells, in particular Bregs, have immunosuppressive functions in numerous inflammatory diseases, including food allergy. Bregs produce several immunosuppressive cytokines, such as IL-10, TGFβ β, and TSP1 which are involved in repression of T-cell mediated inflammation, in enhancing of Treg function, in inducing tolerogenic DCs, and in altering the phenotype of other local B cells.^26^ Bacterial signals can influence Breg phenotype, through interaction with SCFAs,as butyrate, which can also influence IL10 production in Bregs. In particular, butyrate can induce an increasing of the quantity of the serotonin-derived metabolite 5-hydroxyindole-3-acetic acid (5-HIAA), which binds to the Aryl Hydrocarbon receptor (AhR).^27^ AhR acts a transcriptional regulator for Bregs, increasing the production of IL-10 and suppression of proinflammatory cytokines, as TNFα and IL-6.^26^

## Probiotics and AIT

In the last few years, some studies have evaluated the possible use of probiotics, in light of their stimulation of innate immunity via toll-like receptor activation, as adjuvants in AIT (Table 1).

**Table 1:** Results of the use of probiotics as adjuvants in AIT

Human studies				
Authors	Study population	AIT	Probiotics	Outcomes
Jerzynska J et al.	100 children, ages 5–12 years, sensitive to grass pollen, with AR.	5-grass SLIT 300 IR tablets.	Lactobacillus rhamnosus GG.	In the SLIT-probiotic group, there was a significant CD4^+^CD25+Foxp3+ induction compared with the SLIT group and higher reduction in the percentage of TLR-positive cell group compared with the SLIT-vitamin D group.
Kardani Ak et al.	31 children with mild asthma.	SCIT for HDM.	Nigella sativa; PROBI ® of Medifarma, containing bacteria Lactobacillus acidophilus LA life-5 TM and Bifidobacterium lactis Bb-12TM, vitamin premix (vitamin B10, vitamin B20, vitamin B6, vitamin C), and selenium yeast.	Reported significant differences ACT score before and after the AIT + Nigella sativa treatment, before and after AIT + probiotic treatment, before and after AIT plus Nigella sativa plus probiotic treatment. Correlation test highlighted a significant association between the number of Th17 cells and ACT score. The combination AIT with Nigella sativa and probiotics did not reduce the number of peripheral blood Th17 cells in mild asthmatic children, but can improve the clinical symptom.
Rossi R et al.	30 patients affected by AR.	SLIT (Oralvac Plus®, Allergy Therapeutics LTD, Worthing, UK).	A combination of Lactobacillus rhamnosus LR05, Bifidobacterium lactis BS01 and FOS (Kallergen Th®; Allergy Therapeutics, Milan, Italy)	The average symptom and medication scores were lower for patients treated with combination of probiotics plus SLIT respect to patients treated with either one alone.
Loke P et al.	Children, aged 1–10 years, weighing more than 7 kg, with peanut allergy	Peanut OIT.	Lactobacillus rhamnosus ATCC 53103	Both PPOIT and peanut OIT induced sustained unresponsiveness. Addition of a probiotic might offer a safety benefit compared with OIT alone, particularly in preschool children.
Xu LZ et al.	Patients with HDM-induced AR.	SCIT for HDM (Allergopharma Joachim Ganzer KG; Reinbek, Germany).	Clostridium butyricum (Cb).	Co-administration of Cb significantly enhanced the efficacy of SIT on AR inducing the suppression of total nasal symptom scores, medication scores, serum specific IgE, Th2 cytokines and skin prick test index. There was also reported a significant increase of the regulatory B cell frequency.

Animal studies			
Authors	Animal model	Sensitazion and treatment model	Outcomes
Petrarca C et al.	Thirty pathogen-free BALB/c mice	6 naïve mice (not sensitized/not treated) and the others were immunized by 3 subcutaneous injections of purified rBet v 1 pre-adsorbed onto Al (OH)_3_ in saline. One group was sensitized but not treated. The sensitized groups were administered by oral gavage with:Streptococcus thermophilus (ST);ST expressing rBet v 1 (ST[rBet v 1]); or ST in association with rBet v 1 (ST+rBet v 1).	Oral administration of Streptococcus thermophilus expressing rBet v 1 reported a relevant reduction of the Th2 allergic inflammation in sensitized mice with a shift to Th1 and Treg immune responses. These results appeared to be more intense compared with ST plus Bet v 1 and ST alone.
Van Overtvelt L et al.	BALB/c mice, maintained in a specific pathogen free Environment on an ovalbumin (OVA)-free diet	Sublingual treatment of mice with OVA was administered together with *L. helveticus*/L. casei; some mice were treated with OVA alone or phosphate-buffer saline (PBS).	Sublingual administration in ovalbumin-sensitized mice of *L. helveticus*, but not L. casei, induced a reduction of airways hyperresponsiveness, bronchial inflammation and proliferation of specific T cells in cervical lymph nodes.
Adam E et al.	Murine model of mite allergy	I.n. co-administration of Escherichia coliNissle 1917 strain (EcN) with a recombinant form of the major mite allergen Der p 1(ProDer p 1).	Induction of an allergen-specific IgG2a response; prevention of the production of specific IgE and reduction of IL-5 production.
Aoki-Yoshida A et al.	BALB/c mice	In DO11.10 mice, oral administration of ovalbumin (OVA) alone or OVA with c Lactobacillus gasseri OLL2809 (LG2809).	Oral administration of LG2809 increases the population of plasmacytoid dendritic cellsin the lamina propria, resulting in the enhancement of oral tolerance induction, increasing the production of the effector regulatory T cells.
Kim JH et al.	BALB/c mice	B longum KACC 91563 and E faecalis KACC 91532 were administered to BALB/c wild-type mice, in which food allergy was induced by using ovalbumin and alum	B longum KACC 91563 induces apoptosis of mast cells specifically and alleviates food allergy symptoms.

**Fig. 1:**
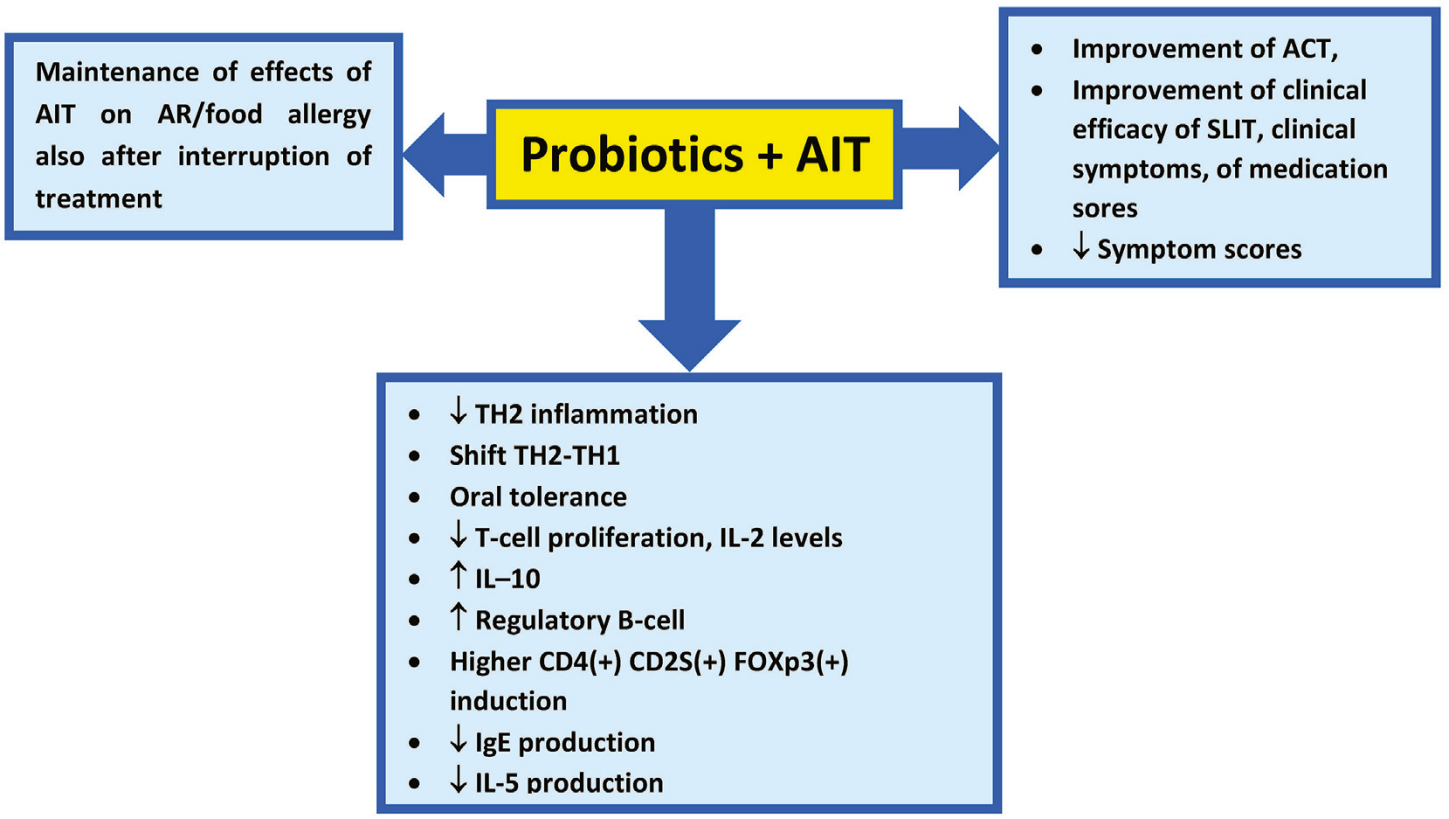
The immunological and clinical effects of “Probiotics +AIT”

Fig. 1 provides an overview of the available evidence on probiotics and AIT combination effects. Considering that some strains of probiotic bacteria can promote TH1 cytokine production and trigger Treg differentiation and activity, as highlighted in mouse models, Petrarca et al reported that their use can prevent or suppress a harmful TH2 response and could be used as adjuvant for AIT, acting with immunomodulatory properties. In particular, the authors reported that the oral use of a strain of *Streptococcus Thermophilus* (ST) expressing Bet v 1, the major allergen of *Betula verrucosa*, can restrict the TH2 allergic inflammatory reaction in sensitized mice, with a shift towards TH1 and Treg allergen-specific immune responses.^28^

Van Overtvelt L et al. studied the immunomodulatory properties of 11 strains of lactic acid bacteria and their capacity to improve SLIT efficacy in a murine asthma model, highlighting that multiple lactic acid bacteria strains have a role in inducing Th1, such as *Lactobacillus casei*, and some have the capacity to support IFN-gamma and IL-10 production, such as *Lactobacillus helveticus*. Sublingual administration in ovalbumin-sensitized mice of L*actobacillus. helveticus*, but not *Lactobacillus casei*, induced a reduction of airways hyperresponsiveness, bronchial inflammation and proliferation of specific T cells in cervical lymph nodes.^29^

Adam E et al reported that in a murine model of mite allergy, the administration of immunotherapy with a recombinant Der p 1 allergen (ProDer p 1) plus a probiotic strain, or E*scherichia coli* Nissle 1917, had a preventive role in the development of allergic symptoms after an airway challenge using mite extracts. Moreover, the authors reported that the coadministration of immunotherapy and probiotics induce an upregulation of allergen specific IgG2a production, a reduction of IgE production and a reduction of IL-5 production by allergen-restimulated splenocytes.^30^

Jerzynska J et al set out to compare the clinical and immunologic efficacy of SLIT given alone or given with probiotic or vitamin D supplementation, reporting a clinical and immunologic effect of probiotic and vitamin D supplementation on SLIT. In particular, the authors enrolled 100 children with grass pollen AR, who participated in a 5-month prospective, randomized, double-blind, placebo-controlled trial, receiving 5-grass SLIT 300 IR tablets with either vitamin D 1000 IU daily supplementation, probiotic, or placebo. The control group was represented by children with allergy who did not qualify for SLIT. In this study, the authors reported a reduction of the symptom-medication score, improvement of lung function, and augmented percentage of CD4(+) CD25(+) Foxp3(+) in children who received SLIT in all the groups compared with the control group. Moreover, in the SLIT-probiotic group, between-group analysis highlighted a relevantly higher CD4(+) CD25(+) Foxp3(+) induction than in the SLIT group and a higher reduction in the percentage of TLR-positive cell group compared with the SLIT-vitamin D group. An increase in CD4(+) CD25(+) Foxp3(+) induction, reduction in TLR-positive cell recruitment and an increase in transforming growth factor β-1 production were independently associated with a better clinical effect of SLIT in children.^31^

Kardani AK et al reported that the use of *Lactobacillus acidophilus* and *Bifidobacterium lactis* induce an improvement of asthma control test (ACT) scores in children with allergic asthma in treatment with AIT for house dust mites (HDM). Some studies reported that the coadministration of *Clostridium butyricum* with AIT in patients with allergic rhinitis and asthma improves the efficacy of AIT, a result that may be related to the increase in allergen-specific regulatory B cells.^32^

Rossi et al, in an observational real-life study, highlighted that supplementation with a symbiotic drug (*Lactobacillus rhamnosus, Bifidobacterium lactis* and *FOS*) during AIT for AR, induces an improvement of the clinical efficacy of SLIT, reporting an improvement of clinical symptoms, a reduction of symptom scores, and an improvement of medication scores, after 2 and 4 months of treatment, without local and systemic adverse reaction.^33^

*Lactobacillus gasseri* OLL2809 (LG2809) fed to a DO11.10-mouse model (carrying a T cell receptor transgene, Tg (DO11.10) reactive to ovalbumin peptide) together with ovalbumin demonstrated the induction of oral tolerance, with a reduction of T-cell proliferation and IL-2 levels, augmented levels of IL-10 from spleen cells and more plasmacytoid DCs in the lamina propria.^34^ In another ovalbumin-food allergy BALB/c mouse model, the administration of probiotic *Bifidobacterium longum KACC 91563*, but not *Enterococcus faecalis KACC 91532*, via the intragastric route or in food, utilized concomitantly with sensitization, showed an improvement of food allergy symptoms. This result was induced by the apoptosis of mast cells and by production of acetate.^35^

Remembering that in humans the efficacy of SLIT is related to the sensitization level of the single patient and the dose administered during therapy, it should be highlighted that during SLIT treatment, there is a lower proportion of CD4^−^CD8 γδ cells and a higher frequency of CD8^+^CD25+IFNγ+ T cells accompanied by reduced inflammatory responses in the lung.^36^ The efficacy of SLIT may also be influenced by Vitamin D3 administration, which potentiated subcutaneous allergen immunotherapy in mice by accumulating Tregs in the lung in an allergen-driven manner.^37^

For FA, which affects 8% of children and 10% of infants in western countries, the oral route is the safer route for administration of immunotherapy. Egg allergy, which affects 0.5–2.5% of children worldwide, is one of the most common food allergies in children, which can persist or present a delayed resolution during school age, and whose management is based on the avoidance of the food concerned and early recognition and treatment of allergic reactions. Recent international guidelines and national expert societies have suggested oral immunotherapy (OIT) for the treatment of egg allergy. The main goals of OIT are the achievement of desensitization, or the temporary (during the antigen exposure) increase of the reaction threshold, and sustained unresponsiveness, or long-lasting tolerance even after stopping allergen immunotherapy. Tolerance is the permanent resolution of an allergy and represents the optimal goal of AIT.^18^

To obtain this goal, some tolerogenic compounds, known as immune response modifiers (IRMs) that modulate the immune response, have been used in combination with AIT. The probiotic *Lactobacillus rhamnosus* American Type Culture Collection (ATCC) 53103 is an IRM, with the ability to induce oral tolerance, Treg, and Th1 cytokine responses.^38^^,^^39^

Loke P et al postulated that the coadministration of this IRM with a food allergen would induce oral tolerance or sustained unresponsiveness to the culprit allergen. This objective is sustained by their previous study in which they used probiotic *Lactobacillus rhamnosus* ATCC 53103 in concomitance with peanut allergen, inducing sustained unresponsiveness to peanuts after 18 months of treatment. In particular, the authors conducted a probiotic and peanut oral immunotherapy (PPOIT) study (PPOIT-001) analyzing PPOIT in 62 children affected by peanut allergy, using a double-blind placebo randomized trial, for 18 months and obtaining 82% of PPOIT with a remission of peanut allergy compared with only 3.6% of placebo-treated patients. The authors reported that, moreover, 80% of PPOIT-treated patients who presented remission of peanut allergy reported a tolerance to peanut even 4 years after the treatment.^40^ Subsequently, Loke P et al led a first double-blind placebo-controlled randomized trial to evaluate the efficacy of probiotic (*Lactobacillus rhamnosus* ATCC 53103) and egg OIT in inducing desensitization or sustained unresponsiveness (remission) in patients with egg allergy compared with placebo.^18^

Always with the aim to study whether the addition of a probiotic adjuvant improved the efficacy or safety of peanut oral immunotherapy, Loke P et al recently conducted a PPOIT-003, multicenter, randomized, phase 2b trial, in 3 tertiary hospitals in Australia in children with peanut allergy. The children were randomly assigned to receive PPOIT, placebo probiotic (*Lactobacillus rhamnosus* ATCC 53103) and peanut OIT, or placebo for 18 months, being followed for up to 12 months after stopping treatment. The authors reported that both PPOIT and OIT were effective at inducing sustained unresponsiveness and the addition of a probiotic can have a better safety benefit compared with OIT alone.^41^

Xu LZ et al evaluated the action of AIT on AR in association with the use of *Clostridium butyricum*. In particular, the authors enrolled patients with dust mite AR, treated with AIT or/and *Clostridium butyricum. Clostridium butyricum* is a probiotic that modulates airway allergic inflammation, improving the nasal epithelial barrier function. Total nasal symptom scores (NSS), medication scores, serum specific IgE levels and Th2 cytokines levels were used to evaluate therapeutic efficacy. In particular, the authors reported that the use of only AIT in patients with AR induced a reduction of NSS and medication score, and an increase of serum specific IgG4, but did not apparently induce alterations in the serum specific IgE, Th2 cytokines levels and skin prick test index, with a relapse of AR 1 month after stopping AIT, unlike patients treated with both AIT/*Clostridium butyricum*. Indeed, the coadministration of AIT with *Clostridium butyricum* reported an improvement of the efficacy of AIT on AR as highlighted by suppression of NSS, serum specific IgE, medication scores, Th2 cytokines, skin prick test index and an increase of the regulatory B cell frequency. These effects of AIT on AR were maintained also after interruption of the treatment. Butyrate, one of the products of *Clostridium butyricum*, plays a key role in blocking the activation of histone deacetylase-1, inhibiting Igε gene transcription, inducing the expression of IL-10 by antigen specific B cells, inducing the generation of Bregs, inducing antigen specific anergy in both cloned and naive CD4^+^ T cells, and activating p300 and STAT3 in DerpsBCs, which are involved in the production of IL-10. In the same study, the authors assessed the serum level of IFN- γ during treatment, not highlighting differences in the treatment with AIT or/and *Clostridium butyricum*. Moreover, they evaluated the frequencies of the peripheral Tregs and Bregs, by flow cytometry, reporting after treatment for 6 months that the frequency of Tregs in the group treated with placebo was significantly lower than healthy controls; instead, the treatment of patients with either AIT or/and *Clostridium butyricum* only slightly up-regulated the frequency of Tregs. Regarding Bregs, frequency was lower in patients with AR than in healthy patients, highlighting that after 6 months of treatment, frequency was upregulated in the AIT/*Clostridium butyricum* group, but not altered in either the AIT group, or *Clostridium butyricum* group, or placebo group. Therefore, Xu LZ suggested that AIT alone might not modulate the Th2 status and the production of specific IgE in patients with AR, with a key role of probiotics being played in improving immune modulation in allergic conditions. In particular, by associating administration to AIT there is an enhancement of the therapeutic efficacy of AIT.^42^

## Conclusions

Probiotics in *in vitro* and *in vivo* studies let us better understand their immuno-modulating role in allergic conditions, in particular in the modulation of the efficacy of AIT (Fig. 1). The interest in probiotics, which has increased in the last 20 years, is related to evidence that a “healthy” microbiota has a positive role in the development of immune tolerance. However, its alteration is related to many life events, in particular in infancy or childhood, such as prematurity, cesarean section, and nosocomial infections. While the use of probiotics has a role, albeit not yet fully recognized and validated, in modulating and preventing several allergic diseases, the use of probiotics as adjuvants for AIT is intriguing, but further studies are needed to better understand which probiotics should be used and to better study the immunological mechanism involved, also identifying possible biomarkers to be evaluated before and after the start of the treatment.

Balancing the immune system and improving the efficacy and the long-term effect of the treatment are the main strengths of the administration of probiotics during AIT, thus reinforcing the objectives of AIT.

Therefore, amongst the unmet needs in the treatment of allergic disorders, there is the need to perform further clinical studies in adults and in children on the use of probiotics in concomitance with AIT, whose supplementation may represent a novel approach to immunomodulate the immunological effects of AIT, increasing its efficacy to better manage multiple human atopic diseases. More data are needed on specific species, dosage, and optimal duration to obtain the correct immunomodulation and a specific and personalized action for each patient and allergic disorder. There needs to be more focus on the immunological changes and on the long-term effect of the treatment, rather than considering only the immediate clinical efficacy, following standardized protocols, always in agreement with international guidelines.

Furthermore, it is important to highlight that if at the basis of the close link between the microbiome and inflammatory pathologies, such as food allergy, there would seem to be metabolites produced by microorganisms, their identification and modulation could be the right strategy to study and understand how probiotics can positively modulate the outcomes of AIT.

## Abbreviations

AIT, Allergen immunotherapy; AR, Allergic rhinitis; Tregs, Regulatory T cells; SCIT, Subcutaneous immunotherapy; SLIT, Sublingual immunotherapy; DC, Dentritic cells; ROS, Reactive oxygen species; APCs, Antigen presenting cells; TLR, Toll-like receptors; ST, Streptococcus Thermophilus; ACT, Asthma Control Test; HDM, House dust mites; OIT, Oral immunotherapy; IRMs, Immune response modifiers; PPOIT, Peanut oral immunotherapy.

## Acknowledgements

Not applicable.

## Funding

The authors declare no funding for this work.

## Availability of data and materials

Not applicable.

## Authors’ contributions

All authors have contributed to the writing and revision of the manuscript. FF, MC and SG are involved in the Conceptualization, writing, review and editing of the paper. EC and GS are involved in writing and original draft preparation and final editing.

## Ethics approval

Not applicable.

## Consent for publication

All authors agreed to the publication of this work in the World Allergy Organization Journal.

## Declaration of competing interest

The authors declare no funding for this work.
